# Ibogaine/Noribogaine in the Treatment of Substance Use Disorders: A Systematic Review of the Current Literature

**DOI:** 10.2174/1570159X21666221017085612

**Published:** 2023-09-01

**Authors:** Alessio Mosca, Stefania Chiappini, Andrea Miuli, Gianluca Mancusi, Maria Chiara Santovito, Francesco Di Carlo, Mauro Pettorruso, John M. Corkery, Carlos Canessa, Giovanni Martinotti, Massimo Di Giannantonio

**Affiliations:** 1 Department of Neurosciences, Imaging and Clinical Sciences, Università degli Studi G. D’Annunzio, Chieti-Pescara, 66100, Italy;; 2 Psychopharmacology, Drug Misuse and Novel Psychoactive Substances Research Unit, School of Life and Medical Sciences, University of Hertfordshire, Hertfordshire, AL10 9AB, UK;; 3 The Elms Surgery, Watford, Hertfordshire, WD17 4NT, UK

**Keywords:** Ibogaine, noribogaine, substance use disorder, addiction, psychedelics, withdrawal symptoms

## Abstract

**Background:**

Ibogaine and noribogaine are psychedelic substances with dissociative properties naturally occurring in plants of the Apocynaceae family. Research has shown their efficacy in treating substance use disorders (SUD), particularly in opiate detoxification, but their efficacy and toxicity are still unclear.

**Objective:**

This review aims to assess the anti-addictive role of ibogaine and evaluate its side effects.

**Methods:**

A systematic literature review was conducted on the 29^th^ of November 2021 using PubMed, Scopus and Web of Science databases through the following search strategy: (“Ibogaine” OR “Noribogaine”) AND (“SUD” OR “substance use disorder” OR “craving” OR “abstinence” OR “withdrawal” OR “addiction” OR “detoxification”) NOT animal NOT review NOT “*vitro.*” The Preferred Reporting Items for Systematic Reviews and Meta-Analyses (PRISMA) was followed for data gathering purposes. Research methods were registered on PROSPERO (CRD42021287034).

**Results:**

Thirty-one articles were selected for the systematic revision, and two were considered for analysis. The results were organised according to the type of study: case reports/case series, randomised-controlled trials (RCTs), open-label, survey and observational studies. The main outcomes were related to the anti-addictive effect of ibogaine and its cardiac toxicity. A meta-analysis of side effects was conducted using RevMan 5.4 software, showing a significant risk of developing headaches after ibogaine/noribogaine treatment.

**Conclusion:**

The results show some efficacy of ibogaine in the treatment of SUDs, but its cardiotoxicity and mortality are worrying. Further studies are needed to assess its therapeutic efficacy and actual safety.

## 1 INTRODUCTION

Ibogaine is an alkaloid with hallucinogenic properties derived from the root of Tabernanthe iboga, a shrub found in the rainforests of West Africa. Ibogaine has been used for centuries as an epiphanic sacrament in spiritual celebrations by the Babongo and Mitsogo, peoples of West Central Africa adherents of the Bwiti religion, a cult widespread in Gabon, Zaire, and Cameroun [1]. In Bwiti rituals, ibogaine is taken both for its stimulating effects and for initiation rites to establish contact with ancestors in the spirit world [2]. In religious rituals, it is taken by chewing directly on the roots of the shrub or by swallowing the powder obtained from the bark with water [3]. As a drug, the most standardized formulation is the ibogaine hydrochloride salt which is usually taken orally [4]. It is rapidly metabolized in the liver to noribogaine, its active metabolite with a long half-life in the blood of about 28-49 h [5-7]. At low doses (5 mg/kg body weight), ibogaine has a mild stimulant effect [8]; when used in higher doses, it produces psychedelic effects, including hallucinations and altered states of consciousness such as an intense dream-like state while awake [8, 9]. Ibogaine’s mechanism of action is very complex and not yet fully understood. Studies have shown that it acts on many different neurotransmitter systems simultaneously. Indeed, both ibogaine and noribogaine can competitively block the dopamine active transporter (DAT) while they noncompetitively inhibit the serotonin transporter (SERT) [10, 11], showing a high potency as serotonin reuptake inhibitors [5, 12]. The effects of ibogaine on mu-opioid receptors are controversial, with only a few studies supporting mu-opioid agonism [7, 13], while *in vitro* studies have found only partial agonism [14, 15]. Its hallucinogenic and psychotropic effects appear to be due to ibogaine's agonistic action on the 5HT2A serotonin receptor [16, 17] and noribogaine kappa-opioid receptor agonism [18, 19]. Furthermore, ibogaine is an antagonist of N-methyl-D-aspartate (NMDA)/glutamate [20, 21], binds at σ-1 and σ-2 receptors and is an antagonist of α3β4 nicotinic acetylcholine receptor (nAChR) [22] upregulating glial cell line-derived neurotrophic factor (GDNF) in the ventral tegmental area (VTA) [23, 24]. Some studies suggest that ibogaine can act on gene expression by reversing the effects of opiates, returning receptors to a pre-dependent condition [25]. It also appears to reverse the cycles and pathways of addiction in the brain [23]. Indeed, in the 1960s, Howard Lotsof, a lay experimenter addicted to heroin, discovered, based on his serendipitous experience, that single doses of ibogaine could decrease craving and prevent withdrawal symptoms in opioid-dependent subjects [5, 26]. Subsequently, several studies tested ibogaine for the treatment of substance abuse in humans, particularly opioid addictions suggesting that it was able to reduce opioid craving, eliminate signs and symptoms of opioid withdrawal, and aid in the transition to abstinence [5, 12]. It has also proven to be effective in treating cocaine, methamphetamine, nicotine, and alcohol addiction [12]. The potential anti-addictive properties of ibogaine were confirmed in animal models showing ibogaine’s efficacy in attenuating substance-seeking behaviors and opioid self-administration and alleviating opioid withdrawal symptoms [16, 27-29]. Despite these premises, today, ibogaine is a Schedule I drug in the United States, defined as having no currently accepted medical use and a high potential for abuse [30]. It is also illegal in Belgium, Denmark, Finland, France, Hungary, Italy, Norway, Poland, Romania, Sweden, Switzerland, and Turkey [8, 31, 32], while in Australia, Canada, and New Zealand, it is a prescription drug for the reduction or elimination of addiction to opiates [31]. A substantial “medical subculture” has sprung up around ibogaine, and it is currently used to treat addiction in clandestine practices or clinics in countries where it is legally prescribed (New Zealand and Canada) or where its use is unregulated (*e.g*., Mexico, Thailand, the Netherlands) [12, 33]. Ibogaine has never been shown to be effective for detoxification in Food and Drug Administration (FDA) or European Union drug-approved clinical trials [34, 35] due to consistent concerns about cardiovascular safety and potential drug interactions [6], so further research development has been prevented [36, 37]. *Aims of the study:* The purpose of this review was to investigate the role of ibogaine in the treatment of SUD, craving, and withdrawal syndromes by conducting a literature review of current related knowledge and by conducting a meta-analysis of side effects to identify what may be the most effective dosages and possible risks and understand whether ibogaine may offer therapeutic advantages over current treatments.

## 2 MATERIALS AND METHODS

### 2.1 Systematic Review Procedures

A systematic electronic search was performed on the 29^th^ of November 2021 on the main scientific databases (supplementary material). The systematic review was structured per the 2020 PRISMA [38] and PROSPERO guidelines [39]. Identified studies were assessed at the title/abstract and full-text screening against eligibility criteria.

### 2.2 Data Synthesis Strategy

**Fig. (1):**
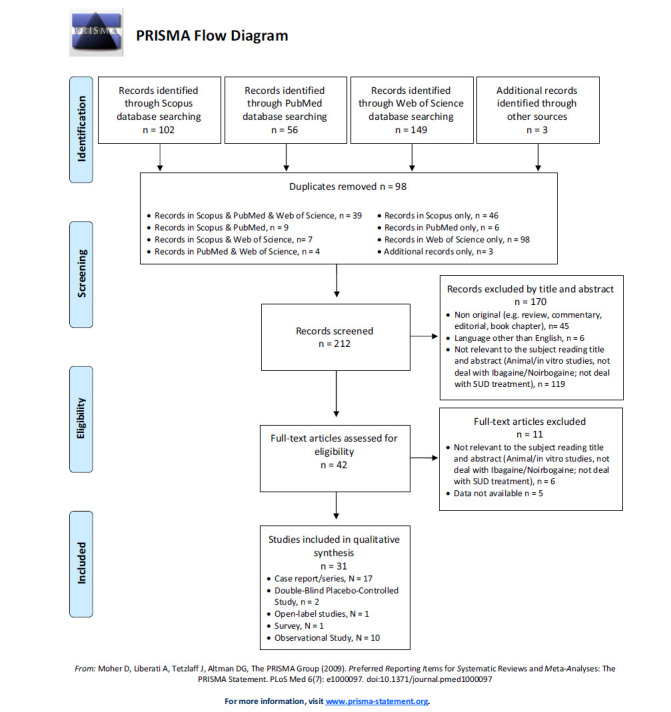
PRISMA flow diagram.

Data were extracted independently by n = 2 investigators (AMo, and MCS) supervised by SC, AM and MP, and doubtful cases were discussed by professors GM, MdG and FS. The exclusion criteria for both selection phases were: 1) non-original studies (*e.g*., review, commentary, editorial, book chapter); 2) non-full-text articles (*e.g*., conference abstract); 3) language other than English; 4) animal/*in vitro* studies; 5) articles not dealing with ibogaine/noribogaine; 6) articles not dealing with SUD treatment. Removing duplicate articles (n = 98) from a total of 310 papers (PubMed = 56; Scopus = 102; WoS = 149; additional records identified through other sources = 3), a total of 212 records were screened, and, of these, 119 were irrelevant to the subject reading title and abstract (animal/*in vitro* studies, not dealing with ibogaine/ noribogaine; not dealing with SUD treatment), 6 were not written in English, and 45 were non-original articles (*e.g*., review, metanalysis, commentary, letter to the editor without data available, book chapter). Of the 42 full-text articles assessed for eligibility, 6 did not match the inclusion criteria for our review, and 5 were unavailable. Finally, 31 articles were included (Fig. **1**). All these research methods were registered by PROSPERO (identification code CRD42021287034).

### 2.3 Risk of Bias

The risk of bias assessment was measured independently by AMo and AM using the Cochrane risk of bias tool [40] only for Double-Blind Randomized Controlled Trials (DBRCT). This result was discussed with MP, GM and MdG.

### 2.4 Quantitative Analysis

The main issue was calculating the risk of developing common adverse events after using ibogaine to treat SUDs. The meta-analysis was performed using Review Manager Software v 5.4 [41], comparing ibogaine with any other treatment in DBRCT studies. Given that these types of DBRCTs are very rare and to include the studies with an event frequency of zero, a risk difference (RD) and not a risk ratio was applied [42, 43]. The RD of the adverse events for each article was calculated and, therefore, computed together, obtaining a Fixed Effect with a 95% confidence interval (CI). Statistical significance was set for values of *p* <0.05. We used I^2 to calculate the heterogeneity of the studies: I^2<30% low heterogeneity; 30% <I^2<60% moderate heterogeneity; 60% <I^2<75% substantial heterogeneity; I^2> 75% considerable heterogeneity [44]. The meta-analysis was calculated for each adverse event identified. A funnel plot of study effect sizes was visually inspected for asymmetry to assess potential publication bias.

## 3 RESULTS

### 3.1 Case Report/Case Series

Of the 31 selected articles (Table **1**), seventeen were case reports/case series [4, 45-60]. Subjects were all male except for two case reports [46, 49] and a few case series [4, 53, 58, 60] taken together; the subjects of the case reports were a total of 33 males and 10 females; subjects' ages ranged from a minimum of 25 [54] to a maximum of 61 years [50]. With regard to the substance-related diagnoses detected, eleven articles dealt with opioid/heroin use disorder [4, 45, 47, 49, 51-54, 56, 58, 60]; two articles with both an opioid (heroin and/or methadone) and a cocaine use disorder [46, 59]; two papers recorded an alcohol use disorder [48, 57], and a polysubstance use disorder [53, 55]; finally one article did not indicate the substance-related diagnosis [50]. In most articles, psychiatric comorbidity was not mentioned; however, attention deficit hyperactivity disorder (ADHD) [47, 49, 53], post-traumatic stress disorder (PTSD) [48], and depression [50] were reported. Ibogaine was always orally consumed, with dosages ranging from a minimum of 50-525 mg [4] to a maximum of 4 g [52], although in several cases, the dosage was non-specific or not reported [53, 55]. One article reported the intake of 1, 550 mg on day 1 and subsequent inhalation of 5-MeO-DMT on day 3 [48]. Regarding the setting, eleven articles reported intake in a home/clandestine context [45, 46, 59, 50-57], while only five were in medical/clinical settings [4, 47-49, 60]. One article reported consumption in both settings [58]. Among those that reported home/clandestine context, five articles reported the online purchase of the drug [45, 46, 51, 52, 56]. Most articles did not report concomitant use of other drugs or substances. Only two articles reported the intake of oral therapy by the subjects [4, 53], while some reported the intake of other abusive substances during ibogaine treatment [4, 47, 52, 53, 55, 57, 58]. The commonest outcome was the anti-addiction effect of ibogaine in terms of a decrease in craving and/or reduction in self-administration and/or substance use cessation [4, 47-49, 60] and withdrawal symptoms mitigated/cessation [47, 60]. Death was also a highly represented outcome [45, 50, 52, 54, 55, 57, 58] followed by significant cardiac adverse effects [46, 51, 56, 59]. One article reported a case of mania following ibogaine ingestion [53]. Finally, in terms of adverse events recorded, the most significant reported ones were cardiac adverse events, many of which resulted in death as the main outcome. Specifically, they were: i) torsade de pointes/arrhythmia/polymorphic ventricular tachycardia/ ventricular flutter/QTc alteration/bradycardia/asystole, [4, 45-47, 50-52, 56]; ii) hallucination/psychosis/psychotic experience [46, 47, 53, 54]; insomnia [53, 60]; iii) irreversible ataxia/muscle spasms [47, 54]; iv) manic symptoms [53, 60] v) and various non-specific internal symptoms *e.g*. ataxia, nausea, diarrhoea [46, 47, 49, 52-54, 59, 60]. Also reported were: one case of hallucinogen-persisting perception disorder (HPPD) [47], suicidal ideation [53], central nervous system depression [46] and one case of respiratory difficulty followed by cardiopulmonary arrest [54].

### 3.2 Double-blind Placebo-controlled Study

Two articles were double-blind, placebo-controlled studies [61, 62]. The first treated 27 adult subjects, 21 males and 6 females (mean age was 41.2 yrs) suffering from opioid use disorder. Ibogaine was taken orally at a dosage of 60 to 180 mg and was compared with a placebo. Ibogaine was taken in a clinical setting, decreased opioid withdrawal ratings were reported (not statistically significant compared to placebo), and side effects were headache and nausea [61]. The second treated 20 male subjects aged 18-64 years suffering from cocaine use disorder with oral capsules containing 1800 mg ibogaine compared to a placebo. Ibogaine was taken under medical supervision and reduced symptoms of cocaine dependence. Side effects were visual hallucinations without cardiovascular events [62].

### 3.3 Open-label Studies

An open-label study [6] reported 27 subjects diagnosed with opioid or cocaine use disorder, treated using ibogaine orally at doses of 500 to 800 mg, showing decreased depressive symptoms and craving.

### 3.4 Survey

One article was a survey [63] describing 27 subjects with an average age of 35 years (gender not reported) suffering from SUD (alcohol or drugs-not specified) taking ibogaine in a home/clandestine setting at an unspecified dosage and mode of intake. They reported reduction in withdrawal symptoms and cravings. Regarding side effects, hallucinations and other non-specific side effects (*e.g*., dizziness, nausea, and diarrhoea) were reported.

### 3.5 Observational Study

There were ten observational studies [33, 64-72]. Two articles reported the same data, so they were treated together [33, 72]. Apart from one article that treated 14 subjects, of which 7 were male, and 7 were female [70] and another where the sex of the subjects was not specified [64], in all other articles, the sample was male-prevalent. The mean age ranged from 27.3 ± 4.7 [33, 72] to 38 ± 4.8 [65]. Most reported a heroin/opioid use disorder, except for one article reporting the use of alcohol, cannabis, cocaine, and crack [71]. No articles reported psychiatric comorbidities. Regarding the route of administration, it was all oral except in one article where it was not reported [66], while the dosage ranged from a minimum of 1 mg/kg [67] to a maximum of 31.4 ± 7.6 mg/kg [65]. One article reported the intake of 200 mg capsules [70], and one case where it was not reported [66]. The setting of intake was always medical/clinical except for one case where it was home/clandestine [33, 72]. Concomitant drugs were not reported except in one article where several substances of abuse were reported [71]. All the studies reported a significant anti-addiction effect (decrease in craving and/or reduction in self-administration and/or substance use cessation) [33, 64-72] and withdrawal symptoms mitigated/cessation [64, 65, 67, 69, 70]. One study reported one death [33, 72], one case of elevated mood [65] and, one case of decreased depressive and anxious symptoms, and increased subjective well-being [69]. Four other studies reported no adverse effects [66-68, 70], four others nausea and vomiting [33, 69, 71, 72], two cases of ataxia [64, 69], one case of hallucination [65], one case QTc alterations and bradycardia [64] and finally others and non-specific [33, 65, 69, 71, 72].

### 3.6 Meta-analysis

**Fig. (2):**
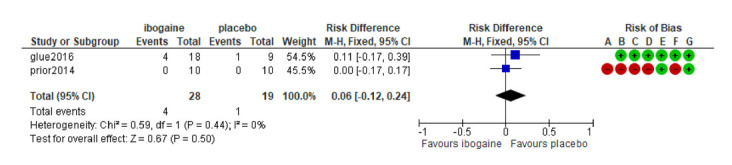
Forest plot: Risk of developing nausea after ibogaine treatment in SUD.

**Fig. (3):**
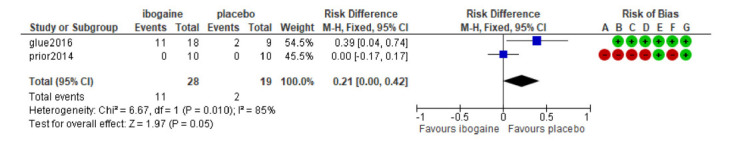
Forest plot: Risk to develop visual impairment after ibogaine treatment in SUD.

**Fig. (4):**
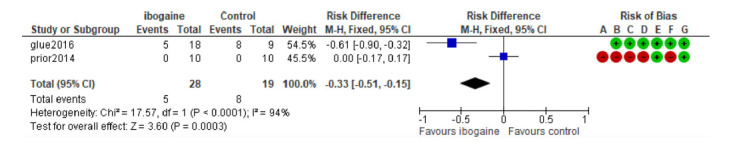
Forest plot: Risk to develop a headache after ibogaine treatment in SUD.

The meta-analysis of the two included studies (ibogaine treatment n subjects = 28; placebo treatment n subjects = 19) showed no significant result about the risk of developing nausea (RD = 0.06; CI 95% = -0.12 to -0.24; *p* = 0.5; I^2 = 0%) and visual impairment (RD = 0.21; CI 95% = 0.00 to 0.42; p = 0.05; I^2 = 85%) after treatment with ibogaine. A small significative risk to develop headache after ibogaine treatment was detected (RD = -0.33; CI 95% = -0.51 to -0.15; *p* < 0.001; I^2 = 94%). (Figs. **2**, **3** and **4**).

### 3.7 Risk of Bias and Publication Bias

**Fig. (5):**
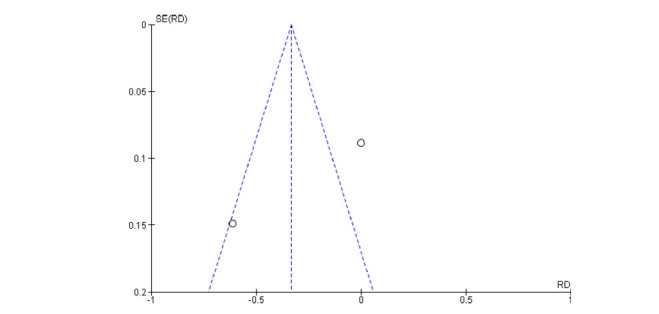
Funnel plot for publication bias (headache).

**Fig. (6):**
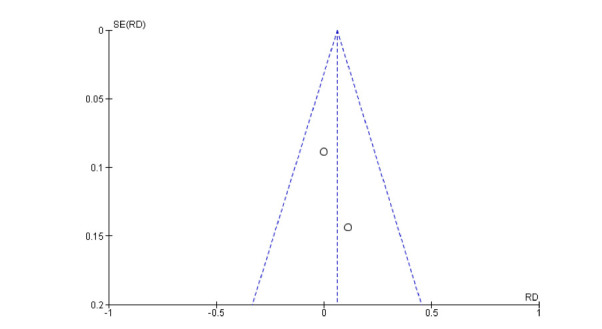
Funnel plot for publication bias (nausea).

The results of the risk of bias assessment reveal a good quality of the reported data in both the articles included only for the Incomplete outcome data item. (Figs. **5** and **6**) The inspection of the funnel plot of the RD of the studies included (Fixed Effect) suggested symmetry of the studies included with a better distribution for the nausea adverse event.

## 4 DISCUSSION

This study is the first review systematically analyzing the use of ibogaine as a treatment for SUD. Although only two double-blind placebo-controlled studies have emerged [61, 62], our results seem to confirm preclinical studies on animals that showed the anti-addictive properties of ibogaine, reducing craving and self-administration of opioid, alcohol and cocaine and its effectiveness against opioid withdrawal [34]. In fact, the anti-dependence effect and the effect on withdrawal symptoms were the most represented in our results. Although the mechanism of action, ibogaine can decrease craving and self-administration of substances is still unclear. Likely, the anti-dependence effect of ibogaine in different classes of substances of abuse is explained by its complex mechanism of action on different receptors [5, 8]. One of these could be its agonistic action on the serotonin 5HT2A receptor [73], linked to its hallucinogenic and psychedelic effects [37] and the epiphanic visionary experience that could be responsible for its therapeutic properties [65, 74]. In recent years, the scientific community's interest in using psychedelics in treating mental disorders has grown, and there is now talk of a 'renaissance of psychedelic medicine' [75, 76]. Several studies are investigating the potential role of these substances in the treatment of SUD and depression [77], in particular, ayahuasca [78], psilocybin [79], ketamine and esketamine [80]. In fact, a recent review on the use of psychedelics in treating psychiatric and addictive disorders [81] showed that in SUD subjects, the intensity of the acute psychedelic experience was the main predictor of the response to the drug. This result was also suggested for ibogaine, as responders to the drug had more spiritually significant experiences than non-responders with improved insight into the cause of their addiction [81]. Another of ibogaine's possible mechanisms of action in opioid addiction is its ability to cause a rapid reset of mu opioid-expressing neurons in the brain's reward centres [82]. Moreover, from studies in animal models, it appears that ibogaine and noribogaine may lead to an activation of the GDNF pathway in the VTA of the brain [23] and could be effective in the treatment of Parkinson's disease [83]; similarly, studies have shown that noribogaine led to a reduction in opioid intake and a concomitant increase in GDNF RNA expression in the absence of neurotoxicity [84]. Finally, ibogaine can modify the expression of the brain-derived neurotrophic factor (BDNF) [83], and it appears that it may act on gene expression by reversing the effects of opioids, returning receptors to a predependent state [25]. Unfortunately, in addition to the anti-dependency effect, our results showed a high risk of mortality [33, 45, 50, 52, 54, 55, 57, 58, 72] mainly related to the cardiotoxicity of ibogaine; these results overlap with a recent systematic review that specifically analysed the adverse effects of ibogaine (CITA). In fact, Ona *et al.*, reported QTc prolongation, tachycardia, hypotension, wide QRS complex and Torsede de Pointes among the main acute adverse effects of taking ibogaine, including some cases of fatalities. Several preclinical studies have shown that ibogaine acts at the level of voltage-dependent cardiac ion channels, such as hERG potassium channels, Nav1.5 sodium channels, Cav1.2 calcium channels, and L-type calcium channels, by altering repolarisation of the cardiac action potential ventricular cardiomyocytes [85-87]. How these alterations lead to death in humans has yet to be investigated further, only one study reported that all fatalities were associated with high potassium and magnesium imbalances [86]. Despite this, it is likely that subjects in treatment had not previously been screened to exclude any cardiac disease, electrolyte imbalances or QT-prolonging drugs, which were not reported by most studies. In fact, as our results revealed, most deaths occurred in a home/clandestine intake setting [33, 45, 52, 54, 55, 57, 58, 72] where adequate medical monitoring capabilities and cardiac support were probably not available. Furthermore, we do not know what methods were used to extract ibogaine and its actual purity, which may be a determinant of its toxicity [88], as well as the huge variety of dosages reported in the literature. Therefore, it appears difficult to understand which therapeutic index could be considered. It is also likely that ibogaine-related deaths have occurred in unsafe environments with improvised protocols. As other authors have pointed out, given the incomplete information, it is difficult to assess the real cause of ibogaine deaths [45]. Other studies, in fact, suggest that under controlled clinical conditions, the drug is safe and well-tolerated [7]. Unfortunately, there is a lack of specific studies on this subject to date [89]. Moreover, other clinical and psychiatric issues cannot be ruled out, as for the possibility of inducing psychotic experiences [90], Hallucinogen persisting perception disorder HPPD, a disorder characterised by lasting or persistent visual hallucinations or perceptual distortions after the use of hallucinogenic drugs [91], and serotonin syndrome [92], as shown in some reports. Unfortunately, our meta-analysis on side effects could only consider the two Double-Blind Placebo-Controlled Studies from which no cardiac side effects or deaths emerged. In this regard, both studies were conducted in a controlled clinical environment. Our meta-analysis showed no significant results about the risk of developing nausea and visual impairment after treatment with ibogaine, whereas there was a small significant risk of developing headaches. This result confirms the relative safety of ibogaine but contradicts studies suggesting the use of psychedelics (LSD and psilocybin) in treating headaches [93, 94]. The drug is likely to cause secondary headaches but may be effective in cases of primary headaches. Furthermore, not all psychedelics have the same therapeutic effects, and while LSD and psilocybin may be effective in treating headaches, ibogaine may worsen them. Further studies are needed. Although indicative of ibogaine efficacy in SUD, findings are heterogeneous and do not allow us to establish a protocol to ensure an optimal therapeutic effect that reduces side effects. The results of the two double-blind placebo-controlled studies are mixed: in the first, although a decrease in opioid withdrawal ratings was reported, this was not statistically significant. Perhaps the limitation is the low dose (60 to 180 mg), which, together with the type of patients - notoriously difficult to treat - may have led to the negative result [61]. In the other study [62], the reduction in cocaine dependence was significant, but the dose was 10 times higher (1800 mg), suggesting that dosages must be consistent to achieve the desired therapeutic effect. Unfortunately, the current legal status of ibogaine has severely limited its research [12]. In line with our findings, typical clinical use for addiction treatment involves ingesting ibogaine hydrochloride salt (ibogaine HCl) at a dosage of 15-20 mg/kg of the patient's body weight. This is in line with Lotsof's manual [95], which guides the best protocol. The Global Ibogaine Therapy Alliance (2015) has also drawn up guidelines for using ibogaine in detoxification [8]. Other authors propose treatment in psychoanalytic clinical settings followed by psychoanalytic psychotherapy for up to 2-3 years or intensive one-week treatments [25]. There is no accurate account of the prevalence of ibogaine's current use, and estimating it is very difficult [52]. In an attempt to estimate the true number of ibogaine users in medical and clandestine settings, a 2008 study [12] analyzed data available from treatment centres, the web and the academic literature and attempted to estimate the extent of 'hidden' populations. This study estimated that those who received ibogaine treatment in the five years to February 2006 outside the West African context could be around 4300-4900 individuals. The figure has likely increased substantially since then [8]. Even more worrying is, with a simple web search, the number of clinics offering ibogaine treatment for opioid addiction and the sites selling it [8]. Unfortunately, during the first half of the 1990s, following the death of a patient in the Netherlands, trials of ibogaine in humans were all stopped. The National Institute on Drug Abuse (NIDA) chose not to fund the proposed phase I/II clinical trials [96], and the FDA blocked the Phase I clinical trial on the use of ibogaine in recently abstinent patient volunteers [37, 97]. However, as the knowledge of ibogaine’s mechanisms of action and its metabolism has increased in recent years, further studies are needed to understand the most suitable patients for ibogaine treatment by creating strong protocols and well-defined inclusion and exclusion criteria to minimise adverse effects. Potential prolongation of QT intervals should not automatically be an obstacle to therapy, which, if conducted under close medical observation, with constant monitoring and management of any cardiac arrhythmias, may prove acceptable [8, 86]. New clinical trials are under development [98-101] and may dispel doubts about the real effectiveness and toxicity of the drug. While ibogaine may ultimately be considered an effective therapy in SUD but with worrying cardiotoxicity, synthetic molecules derived from it, such as 18-methoxycoronaridine (18-MC) [16] and tabernanthalog (TBG) [102], could be very promising. Both molecules were developed independently to create a medicine with the anti-addictive efficacy of ibogaine but without its adverse effects and could prove to be revolutionary drugs for the treatment of addiction [16, 103].

## CONCLUSION

Although the results show some efficacy of ibogaine in treating SUD, its cardiotoxicity and mortality are of concern. Unfortunately, only two Double-Blind Placebo-Controlled Studies emerged from our investigation. The lack of adequate controlled clinical trials does not allow a definitive answer about the therapeutic efficacy of ibogaine and its safety. New studies in double-blind, randomized clinical settings with placebo and metabolism screening are needed together with drawing up protocols for observation and administration of ibogaine and inclusion and exclusion criteria to define with certainty its level of efficacy and toxicity and to assess its risk/benefit ratio.

## LIMITATIONS

The main difficulty regarding the literature on the use of ibogaine in treating SUD concerns its heterogeneity due to the lack of controlled clinical trials. In fact, most of the selected articles were case reports/case series or observational studies. Only two double-blind studies emerged, a limitation in determining the therapeutic efficacy of ibogaine and the meta-analysis results, which would have benefited from a larger number of studies. This is probably due to the legal status of ibogaine, the administration of which is banned in most Western countries and whose trials have been blocked by the NIDA and the FDA. Many cases analyzed occurred in the home or clandestine settings, making accurate clinical, intervention and outcome assessment impossible. For the same reason, there are no shared guidelines and/or consistent administration protocols, making a comparison between studies difficult.

## Figures and Tables

**Table 1:** Main findings of retrieved studies.

References	Population	Mean Age in Years; (SD for Non-case Report Studies)	Substance-Related Diagnosis	Psychiatric Comorbidity	Dosage, Roa, Source Where Ibogaine was Acquired	Context of Use	Concomitant Drug	Outcome	Adverse Events Recorded
**Case Reports/Case Series**									
Aćimović *et al.*, 2021 [45]	N=1; M	27	SUD (heroin)	ND	Oral, *T. Iboga* root bark powder bought online.	Home/ clandestine	ND	Death	Torsade de pointes
Wilson *et al.*, 2020 [4]	N=2; M=1, F=1	35; 34	SUD (opioid)	ND	Oral; administered by two unregulated private clinics 1^st^ patient: 50-525 mg Ibogaine HCL + 100-500 mg Iboga (variable doses over 6 days); 2^nd^ patient: from 1,670 to 725 mg Ibogaine HCL (5 administrations over 4 months).	Medical/ clinical	1^st^ patient: tobacco, illicit oxycodone, diazepam 10 mg; 2^nd^ patient: alcohol, methamphetamine, illicit opioids (*i.e*., fentanyl/heroin) *via* inhalation/smoking, in addition to prescribed sustained release oral morphine	Opioid use cessation for 3 and 2 years; withdrawal symptoms mitigated	QTc prolongation, bradycardia
Grogan *et al.*, 2019 [46]	N=1; F	34	SUD (heroin and cocaine)	ND	Oral; 2 g of Ibogaine powder, bought online.	Home/ clandestine	ND	Significant cardiac and neurologic adverse effects	Torsade de Pointes, QT-segment prolongation, cardiac dysrhythmias, hallucinations, seizure-like episodes, and central nervous system depression
Barsuglia *et al.*, 2018 [48]	N=1; M	31	SUD (alcohol)	PTSD	Oral (1550 mg, 17.9mg/kg of Ibogaine HCL) on day 1; inhalation of 5-MeO-DMT (≈5-7 mg) on day 3. Administered by a treatment facility.	Medical/ clinical	ND	Improvement in mood, cessation of alcohol use, and reduced cravings at 5 days post-treatment. Effects sustained at 1 month, with a partial return to mild alcohol use at 2 months	Dream-like visions, including content pertaining to his alcohol use and resolution of past developmental traumas
Knuijver *et al.*, 2018 [47]	N=1; M	31	SUD (heroin)	ADHD	Oral 700 mg (10 mg/kg, single dose of Ibogaine HCL). Administered by a high-care medical facility.	Medical/ clinical	Tobacco	Cessation of morphine, no withdrawal symptoms. Relapse in heroin use. HPPD after 48 h, restarting of opioid substitution therapy	“Oneirogenic,” experience for 5 hours, QTc-prolongation, mild drop-in heart rate and blood pressure, mild ataxia, HPPD
Steinberg *et al.*, 2018 [50]	N=1; M	61	ND	Depression	Oral; ≈ 5.6 g (65-70 mg/kg, single dose). Ibogaine capsules administered by a holistic, naturopathic clinic without a medical prescription.	Home/ clandestine	ND	Cardiac arrest from significant cardiac adverse effects	Massive QT prolongation and ventricular flutter
Cloutier-Gill *et al.*, 2016 [49]	N=1; F	37	SUD (heroin)	ADHD	Oral; a total of 2,300 mg (32 mg/kg) over 4 days treatment. Ibogaine HCL is administered by an Addiction Outpatient Clinic.	Medical/ clinical	ND	The patient described an eye-opening opportunity, giving her emotional strength to attempt and sustain abstinence	Transitory side effects such as weakness, dizziness, and diaphoresis
Hildyard *et al.*, 2016 [51]	N=1; M	39	SUD (heroin)	None	Oral; 2 g of Ibogaine bought online.	Home/ clandestine	None	Significant cardiac adverse effects	QT prolongation and 7 additional episodes of polymorphic ventricular tachycardia that were successfully terminated with electrical treatment
Meisner *et al.*, 2016 [52]	N=1; M	40	SUD (heroin)	ND	Oral; 4 g of Ibogaine bought online.	Home/ clandestine	Uncharacterized “booster” bought on the Internet.	Death from cardiac arrest.	Asystole, emesis, pupils fixed and dilated, hypotension, leucocytosis, metabolic acidosis, QTc -prolongation
Marta *et al.*, 2015 [53]	N=3; M=2, F=1	36, 35, 40	SUD (opiates, cocaine, alcohol, psilocybin mushrooms, marijuana)	ADHD	Oral, type and source not specified.	Home/ clandestine	Valproic acid, risperidone, quetiapine, olanzapine, methadone	Mania following the use of ibogaine. Improvement in mania symptoms after treatment (N=1)	Insomnia, irritability, grandiose delusions, aggression, impulsivity, psychomotor agitation, emotional lability, hallucinations, tangential speech, and suicidal ideation
Jalal *et al.*, 2013 [54]	N=1; M	25	SUD (heroin)	ND	Oral; 2.5 g; type and source not specified.	Home/ clandestine	ND	Death from cardiopulmonary arrest and multi-organ failure	Irreversible ataxia and muscle spasms, hallucinations, weakness, fever, and urinary retention. Then developed respiratory difficulty followed by cardiopulmonary arrest
Mazoyer *et al.*, 2013 [55]	N=1; M	27	SUD (alcohol, cannabis, psilocybin, LSD, benzodiazepine, crack, cocaine, amphetamine, ecstasy, morphine, and heroin)	ND	Oral; “a teaspoon” of powdered iboga root, administered during a non-medical detoxification program.	Home/ clandestine	Methadone and diazepam	Fatal	Death after 12 ibogaine mixed overdose, with ibogaine as the toxic principle, in association with methadone and diazepam
O’Connell *et al.*, 2013 [56]	N=1; M	33	SUD (heroin)	None	Oral; ≈ 3.832 g, Ibogaine capsules bought online.	Home/ clandestine	ND	Significant cardiac adverse effects	Transient prolonged QT intervals during the period of ibogaine intoxication, absence of electrolyte abnormalities or other medication effects
Papadodima *et al.*, 2013 [57]	N=1; M	52	SUD (alcohol)	ND	Oral; concentration of 2 mg/L of blood, not specified type, administered by a non-medical “therapist.”	Home/ clandestine	Silene capensis	Death (comorbidity of coronary disease and serious liver disease)	**-**
Alper *et al.*, 2012 [55]	N=19; M=15, F=4	39.1 ± 8.6	SUD (opioid, N=15)	ND	Oral; 14.3 ± 6.1 mg⁄kg. Ibogaine HCL (N=14); Alkaloid extract (N=2); dried root bark (N=2); brown powder (N=1). Administered by a treatment facility or individually procured.	Both home/ clandestine and medical/ clinical	Commonly abused drugs (including benzodiazepine, cocaine, opiate, and methadone) (N=8)	Death (mainly cardiovascular causes): estimated mean interval from ibogaine ingestion to death =24.6-21.8 h; range=1.5-76 h (N=18)	Advanced pre-existing medical comorbidities, which were mainly cardiovascular, and⁄or one or more commonly abused substances, explained or contributed to the death in 12 of the 14 cases for which adequate post-mortem data were available
Pleskovic *et al.*, 2012 [59]	N=1; M	33	SUD (cocaine, heroin and methadone)	ND	Oral; 600 mg; type and source not specified.	Home/ clandestine	ND	Significant cardiac adverse effects	QTc-interval prolongation (for 9 days) and multiple ventricular fibrillation/ tachycardia; loss of consciousness after a vagal maneuver
Sheppard, 1994 [60]	N=7; M=5, F=2	29.29 ± 5.62	SUD (heroin/ opioid)	ND	Oral; 700-1800 mg of Ibogaine HCL, administered under medical supervision.	Medical/ clinical	ND	No withdrawal symptoms; at 700 mg dose: relapse in drug abuse after 2 days (N=1); at 1,000 mg dose or more: relapse after some week (N=2); Intermittent heroin use (N=1); drug- free 14 weeks or more (N=3).	Slight nose flood, sweating, and cold sensations sometimes (24-38 h). Increased energy, appetite, and a reduced requirement for sleep for several weeks. Physical side effects: weight loss, extreme sensitivity to (red) colour and sound, ataxia, diarrhoea, backache and nausea and vomiting. A strong aphrodisiac effect, completely reversible concentration difficulties, tiredness up to 2 weeks after treatment, sudden loss of coordination and insomnia
**Double-Blind Placebo-Controlled Study**									
Glue *et al.*, 2016 [61]	N=27; M=21	41.2 (Mean age)	SUD (opioid)	ND	Oral: 60 mg (N=6); 120 mg (N=6); 180 mg (N=6) of Noribogaine; placebo (N=9). Administered under medical supervision.	Medical/ clinical	Methadone 25-80 mg/day	Decreased opioid withdrawal ratings (non-statistically significant trend), most notably at the 120-mg dose	Non-euphoric changes in light perception (1h), headache, nausea. Concentration-dependent increase in Qtc (0.17 ms/ng/ mL), with the largest observed mean effect of 16, 28, and 42 milliseconds in the 60, 120 and 180 mg groups, respectively
Prior *et al.*, 2014 [62]	N=20; M=20	18-64	SUD (cocaine)	None according to the exclusion criteria	Oral capsule containing 1800 mg of dried extract of ibogaine at 75% purity (N=10, ibogaine group); placebo capsule (N=10). Administered under medical supervision.	Medical/ clinical	None, according to the exclusion criteria.	In the Ibogaine group, there was a significant reduction of symptoms in the acute stage of cocaine dependence and reduced drug relapse in the chronic stage	Visual Hallucinations during the 72 hours after exposure. No cardiovascular events
**Open-Label Studies**									
Mash *et al.*, 2000 [6]	N=27; M=23	34.6 ± 1.9 (Opioid group); 37.5 ± 2.9 (Cocaine group)	SUD (opioid or cocaine)	Not axis I comorbidity	Oral; 500, 600, or 800 mg of Ibogaine HCL. Administered under medical supervision.	Medical/ clinical	ND	Decreased depressive symptoms and craving (self-reported)	ND
**Observational Study**									
Knuijver *et al.*, 2021 [64]	N=14	48 (Mean age)	SUD (opioid)	None according to the exclusion criteria	Oral; 10 mg/kg of Ibogaine HCL, administered under medical supervision.	Medical/ clinical	ND	Well-tolerated and manageable withdrawal and psychomimetic effects (11/14 did not return to morphine within 24 hours)	Relevant but reversible QTc prolongation, bradycardia, and severe ataxia
Brown *et al.*, 2019 [65]	N=44; M=32 (Mexican group: 25 M; 5 F) (New Zealand group: 7 M; 7 F)	29.0 ± 9.0 (Mexican group); 38 ± 4.8 (New Zealand group)	SUD (opioid)	None according to the exclusion criteria	Oral; 31.4±7.6 mg/kg (N=14 NZ); 22.5±10.1 mg/kg (N=26 Mexican); 9.9±7.8mg/kg ibogaine HCl + 18.7±17.3 mg/kg of iboga rootbark (N=4 Mexican). Administered under medical supervision.	Medical/ clinical	ND	Attenuation of cravings, withdrawals, and acutely elevated mood	“Oneiric state,” hallucinations (auditory, visual, altered perceptions)
Davis *et al.*, 2018 [66]	N=73; M=51, F=22	35 (Mean age)	SUD (heroin 48%, and prescription opioids 52%)	None according to the exclusion criteria	Ibogaine HCL, administered under medical supervision.	Medical/ clinical	ND	One year or more since treatment with ibogaine: 81% (N=59) never used opioids again or decreased use; 19% (N=14) use stayed the same or increased. Some 36% never used opioids again (N=26); 45% decreased use (N=33); 15% had no changes in their opioid use (N=11); 4% increased use (N=3). Overall self-reported positive changes in psychosocial functioning	ND
Malcolm *et al.*, 2018 [67]	N=40; M=24, F=16	31.28 ± 8.38	SUD (heroin, prescription opioids)	None according to the exclusion criteria	Oral; Ibogaine HCL; started with 18-20 mg/kg; then 1-5 mg/kg for the remaining treatment duration (72h) in case of withdrawal symptoms. Administered under medical supervision.	Medical/ clinical	Not in the last week, according to the exclusion criteria	Reduction (79% minimal; 68% mild range) or absence (78%) of opioid withdrawal and craving	ND
References	Population	Mean Age in Years; (SD for Non-case Report Studies)	Substance-Related Diagnosis	Psychiatric Comorbidity	Dosage, Roa, Source Where Ibogaine was Acquired	Context of Use	Concomitant Drug	Outcome	Adverse Events Recorded
Brown and Alper, 2017 [68]	N=30; M=25, F=5	29.0± 9.0	SUD (opioid)	ND	Oral; 1,540± 920 mg ibogaine HCl (+ 1610 ± 1650 mg of *T. iboga* root bark in 5 subjects). Administered under medical supervision.	Medical/ clinical	ND	No opioid use during the previous 30 days at 1 (N=15, 50%) and 3 (N=10, 33%) months. Reduction of drug use at 9 and 12 months (N=12)	ND
Davis *et al.*, 2017 [69]	N=88; M=64, F=22	35 (Mean age)	SUD (opioid)	None according to the exclusion criteria	Oral; 15±5 mg/kg of Ibogaine HCL. Administered under medical supervision.	Medical/ clinical	ND	Total opioids use cessation (30%). Abstinence for at least 1 year (54%) and 2 years (31%). Elimination or reduction of withdrawal symptoms (80%). Reduction of opioid craving (50%); reduction in craving lasting at least 3 months (25%). Decreased depressive and anxious symptoms, increased subjective well-being	Auditory buzzing (tinnitus-like noise), auditory hypersensitivity, ataxia, dissociation, visual tracers, nausea, and vomiting
Noller *et al.*, 2017 [70]	N=14; M=7, F=7	ND	SUD (opioid)	ND	Oral; 200 mg capsules of Ibogaine HCL. Administered under medical supervision.	Medical/ clinical	ND	Reduced opioid withdrawal symptoms. Opioid use cessation or sustained reduced use in dependent individuals over a period of 12 months	ND
Schenberg *et al.*, 2014 [71]	N=75; M=67	34.16 ± 8.33 (male); 29.50 ± 5.31 (female)	SUD (alcohol, cannabis, cocaine, and crack)	ND	Oral. Ibogaine HCL. Men: from 14.81±1.61 to 12.22±3.04 mg/kg (decreasing doses over 4 sessions). 2 had a fifth session (7.5 mg/kg and 14.89 mg/kg). Women: from 12.03±0.85 mg/kg to 11.85±0.21 mg/kg (over 2 sessions). Administered in a private clinic.	Medical/ clinical	Tobacco, alcohol, cannabis, cocaine, crack, opioid, methamphetamines, “acid,” “ecstasy” and prescription substances such as benzydamine and methylphenidate	Abstinence for a median of 5.5 months and for a median of 8.4 months in those treated multiple times	Nausea, ataxia, vomiting, tremors, headaches, and mental confusion
Alper *et al.*, 1999; Alper *et al.*, 2000 [72]	N=33; M=22	27.3 ± 4.7	SUD (opioid)	ND	Oral; 19.3 ± 6.9 mg/kg (range: 6-29 mg/kg), not specified type, administered in non-medical settings.	Home/ clandestine	ND	No opioid withdrawal signs at 24 and 48 hours; no seeking behaviour during the 72-hour post-treatment interval (N=25); drug seeking without withdrawal signs (N=4); drug abstinence with attenuated withdrawal signs (N=2); withdrawal signs and drug-seeking behaviour (N=1); death (N=1, possibly involving surreptitious heroin use)	Insomnia, nausea and vomiting and not pleasant for all psychoactive states. One subject died
References	Population	Mean Age in Years; (SD for Non-case Report Studies)	Substance-Related Diagnosis	Psychiatric Comorbidity	Dosage, Roa, Source Where Ibogaine was Acquired	Context of Use	Concomitant Drug	Outcome	Adverse Events Recorded
**Survey**									
Heink *et al.*, 2017 [63]	N=27	35 (Mean age)	SUD (alcohol or unspecified drugs)	ND	ND	Home/ clandestine	ND	Reduction of withdrawal symptoms and cravings for up to months after treatment	Light-headedness/ dizziness, decreased control of movements in any body parts, buzzing in ears, nausea, vomiting, diarrhoea, feeling physically heavy, movement difficulty, emotional distress, hallucinations

## LIST OF ABBREVIATIONS

ADHD: Attention Deficit Hyperactivity Disorder
BDI: Beck Depression Inventory
BPRS: Brief Psychiatric Rating Scale
CGI: Clinical Global Impression
HPPD: Hallucinogen Persisting Perception Disorder
DSM: Diagnostic and Statistical Manual for Mental Disorders
GDNF: A Glial Cell Line-Derived Neurotrophic Factor
LSD: Lysergic Acid Diethylamide
ND: Non Defined
PANSS: Positive and Negative Syndrome Scale
PTSD: Post-Traumatic Stress Disorder
SCS: Self-Compassion Scale
SD: Standard Deviation
SUD: Substance Use Disorder
VAS: Visual Analogic Scale
VTA: Ventral Tegmental Area
WHODAS: WHO Disability Assessment Schedule
yy: Years
