# Supplementary material for: Ibogaine/Noribogaine in the Treatment of Substance Use Disorders: A Systematic Review of the Current Literature
Source: Curr Neuropharmacol. 2023 Sep 1;21(11):2178–94. doi: 10.2174/1570159X21666221017085612 (PMC10556383; doi:10.2174/1570159X21666221017085612)
Supplement: Supplementary file 1 — Supplementary material is available on the publisher’s website along with the published article. [file CN-21-2178_SD1.pdf]

## Supplementary Material

# Ibogaine/Noribogaine in the Treatment of Substance Use Disorders: A Systematic Review of the Current Literature

Alessio Mosca[1,*], Stefania Chiappini[1,2], Andrea Miuli[1], Gianluca Mancusi[1], Maria Chiara Santovito[1], Francesco Di Carlo[1], Mauro Pettorruso[1], John M. Corkery[2], Carlos Canessa[3], Giovanni Martinotti[1,2] and Massimo Di Giannantonio[1]

*[1]Department of Neurosciences, Imaging and Clinical Sciences, Università degli Studi G. D'Annunzio, 66100 Chieti-Pescara, Italy; [2]Psychopharmacology, Drug Misuse and Novel Psychoactive Substances Research Unit, School of Life and Medical Sciences, University of Hertfordshire, Hertfordshire AL10 9AB, UK; [3]The Elms Surgery, Watford, Hertfordshire WD17 4NT, UK*

A systematic electronic search was performed on 29th of November 2021 on PubMed, Scopus, and Web of Science (WoS) databases. The following search strategies were used, respectively in PubMed and WoS ("Ibogaine" OR "Noribogaine") AND ("SUD" OR "substance use disorder" OR "craving" OR "abstinence" OR "withdrawal" OR "addiction" OR "detoxification") NOT animal NOT review NOT "*vitro*"; in Scopus: (TITLE-ABS-KEY ("Ibogaine") OR TITLE-ABS-KEY ("Noribogaine") AND TITLE-ABS-KEY ("SUD") OR TITLE-ABS-KEY ( "substance use disorder" ) OR TITLE-ABS-KEY ("craving") OR TITLE-ABS-KEY ("abstinence") OR TITLE-ABS-KEY ( "withdrawal" ) OR TI-TLE-ABS-KEY ("addiction") OR TITLE-ABS-KEY ("detoxification") AND NOT TITLE-ABS-KEY ( animal ) AND NOT TITLE-ABS-KEY (review) AND NOT TI-TLE-ABS-KEY ("*vitro*")).

1875-6190/22 $65.00+.00 © 2023 Bentham Science Publishers
